# Intestinal Toxaemia of New Born Due to Retained Meconium

**Published:** 1915-08

**Authors:** 


### ABSTRACTS

---

**Intestinal Toxaemia of New Born Due to Retained Meconium.** P. S. Potter, Syracuse, Pediatrics, June, discusses this interesting subject, with reference to other authorities. As his case developed fever and colicky symptoms on the ninth day, after normal milk stools had appeared, it seems to us questionable whether it belongs in this category, but the subject is worth considering, especially with regard to the modus operandi of early obstipation, and particularly with reference to the toxicity of meconium intrinsically and from bacteria introduced in the first few days of life.

